# High spatial correlation in brain connectivity between micturition and resting states within bladder-related networks using 7 T MRI in multiple sclerosis women with voiding dysfunction

**DOI:** 10.1007/s00345-021-03599-4

**Published:** 2021-01-29

**Authors:** Zhaoyue Shi, Khue Tran, Christof Karmonik, Timothy Boone, Rose Khavari

**Affiliations:** 1 Translational Imaging Center, Houston Methodist Research Institute, Houston, TX, USA; 2 Department of Urology, Houston Methodist Hospital, Houston, TX, USA

**Keywords:** Multiple sclerosis, Voiding dysfunction, Urodynamics, Functional magnetic resonance imaging, Resting states, Brain connectivity

## Abstract

**Background:**

Several studies have reported brain activations and functional connectivity (FC) during micturition using functional magnetic resonance imaging (fMRI) and concurrent urodynamics (UDS) testing. However, due to the invasive nature of UDS procedure, non-invasive resting-state fMRI is being explored as a potential alternative. The purpose of this study is to evaluate the feasibility of utilizing resting states as a non-invasive alternative for investigating the bladder-related networks in the brain.

**Methods:**

We quantitatively compared FC in brain regions belonging to the bladder-related network during the following states: ‘strong desire to void’, ‘voiding initiation (or attempt at voiding initiation)’, and ‘voiding (or continued attempt of voiding)’ with FC during rest in nine multiple sclerosis women with voiding dysfunction using fMRI data acquired at 7 T and 3 T.

**Results:**

The inter-subject correlation analysis showed that voiding (or continued attempt of voiding) is achieved through similar network connections in all subjects. The task-based bladder-related network closely resembles the resting-state intrinsic network only during voiding (or continued attempt of voiding) process but not at other states.

**Conclusion:**

Resting states fMRI can be potentially utilized to accurately reflect the voiding (or continued attempt of voiding) network. Concurrent UDS testing is still necessary for studying the effects of strong desire to void and initiation of voiding (or attempt at initiation of voiding).

## Introduction

Multiple sclerosis (MS) is an autoimmune inflammatory disease, which can impair and disrupt the neural conduction from the brain and spinal cord to other parts of the body. Neurogenic lower urinary tract dysfunction (NLUTD) is a very common symptoms of MS with as many as 90% of patients will experience some degree of voiding dysfunction (VD) and/or incontinence in their life [1, 2]. Neuroimaging via functional Magnetic Resonance Imaging (MRI) is a well-established tool for delineating the activity of brain regions in response to specific tasks [3]. Since the discovery of resting-state brain networks, a powerful approach has been developed to study the intrinsic functional connectivity (FC) between various brain regions [4]. These studies can be further augmented through the use of ultra-high field (UHF) MRI due to the higher signal-to-noise ratios, higher tissue contrast, and increased spatial resolution that UHF provides over conventional lower field MRI [5, 6]. Previous studies using different neuroimaging technologies have investigated the brain’s control over both healthy and neurogenic bladders using several models including the concurrent urodynamics (UDS) testing [7-10]. While these models can provide insight regarding brain activation during the bladder cycle, the physical aspect of invasive catheterization can cause discomfort for participants, and thus limit the potential subject population.

The goal of this study is to evaluate whether resting-state functional connectivity patterns could reflect full bladder versus voiding during simultaneous fMRI/UDS bladder-related networks in female MS patients with VD. Here, we quantitatively compared the functional connectivity in 13 regions of interest (ROIs) belonging to the bladder-related network during the following states of the bladder cycle: ‘strong desire to void’, ‘(attempt at) voiding initiation’, and ‘(continued attempt of) voiding’ with the FC during rest in female MS patients with VD using fMRI data acquired at 7 T and 3 T.

## Methods

### Subjects

Ten adult female ambulatory patients with clinically stable MS for ≥ 6 months and symptomatic NLUTD were recruited for this study, one dropped out due to a recent injury. Nine subjects were included for the analysis (Table 2). Patients with prior slings (midurethral or pubovaginal), bladder/bladder neck suspension operations, or previous bladder reconstruction procedures, such as augmentation cystoplasty, were excluded. Patients who were taking bladder medications at study entry continued to take them and those who were not remained off them throughout the study. Criteria for VD included having a post-void residual (PVR) volume of ≥ 40% of their bladder capacity or having uroflow data falling below 10th percentile on the Liverpool nomogram for maximum urine flow rate in women. All subjects completed a detailed history, physical examination, validated questionnaires, and MRI Safety Screening Questionnaire.

### Concurrent UDS and MRI procedures

Prior to scanning, subjects were asked to spontaneously empty their bladders. MRI-compatible double-lumen 7Fr bladder and rectal UDS catheters were then placed into the patients’ bladder. The tubing was brought out of the scanner room through a waveguide and connected to a UDS system in an adjacent MRI control room. MRI structural and resting-state scans were performed with the patient’s bladder in the empty state. During each concurrent fMRI/UDS scanning, the patients’ bladder was filled with room temperature sterile water at a rate of 50 mL/min. Subjects pressed a button on a hand-held response grid to indicate a strong desire to void, at which point bladder infusion was stopped and subjects were asked to hold for 30 s through instructions projected on a monitor. After 30 s, subjects were given permission to void (through the monitor) onto absorbent pads on the scanner table while lying supine; this time point was marked as “(attempt at) voiding initiation”. A button was pressed again to indicate actual “(continued attempt of) voiding”. Residual of urine was manually aspirated. This urodynamic testing cycle was repeated three to four times for each subject. Complete UDS and PVR during scanning were recorded.

### MRI Data acquisition

Seven subjects’ MR images were acquired on a 7 T scanner (Siemens MAGNETOM Terra) with a 32-channel head coil. Each session included the following scans:

1. 3D T1-weighed MPRAGE anatomical scan (isotropic 0.7 mm spatial resolution);
2. T2*-weighted blood oxygen level-dependent (BOLD) fMRI scan in resting states (TR = 2500 ms, TE = 24 ms, isotropic spatial resolution 1.4 mm, 8 min);
3. Simultaneous T2*-weighted BOLD fMRI during UDS. Acquisition parameters were identical to the resting-state scan; however, scan times were varied due to the voiding process being different in each individual subject.

Due to MR safety regarding the body implants, two subjects’ MR images were acquired on a 3 T scanner (Siemens MAGNETOM Vida) with a 20-channel head coil. Each session included the following scans:

1. 3D T1-weighed anatomical scan (isotropic 0.86 mm spatial resolution);
2. T2*-weighted BOLD fMRI scan in resting states (TR = 2000 ms, TE = 30 ms, isotropic spatial resolution 2.98 mm, 7 min);
3. Simultaneous T2*-weighted BOLD fMRI during UDS as described above.

### Data analysis

FMRI data processing was accomplished using Analysis of Functional NeuroImages (AFNI). The protocol included slice time correction, motion correction, spatial normalization to the Montreal Neurological Institute (MNI) template, and spatial smoothing using a 3 mm full-width-at-half-maximum Gaussian kernel. The resting-state fMRI time series were then temporally bandpass-filtered between 0.01 and 0.1 Hz. After spatial smoothing, individual fMRI activation maps were created using a generalized linear model in MNI space at three urodynamic phases: strong desire to void, initiation of voiding (or attempt at initiation of voiding), and voiding (or continued attempt of voiding). An averaged group fMRI activation map was then created from which areas of significant activation (based on Student’s t-test) were identified (*p* < 0.05). Thirteen regions of interest (ROIs) associated with the bladder cycle were analyzed (Table 1).[9-16] Average fMRI/UDS time series were computed across each ROI and functional connectivity (based on Pearson’s correlation coefficient) between ROI pairs was computed at each bladder-related phase and compared to resting states. The similarity between correlation matrices was quantified with the 2D correlation coefficient.

## Results

### Patient demographics and clinical variables

Table 2 details the patient demographics and their clinical variables.

### Functional connectivity analysis within the bladder-related network

**Fig. 1:**
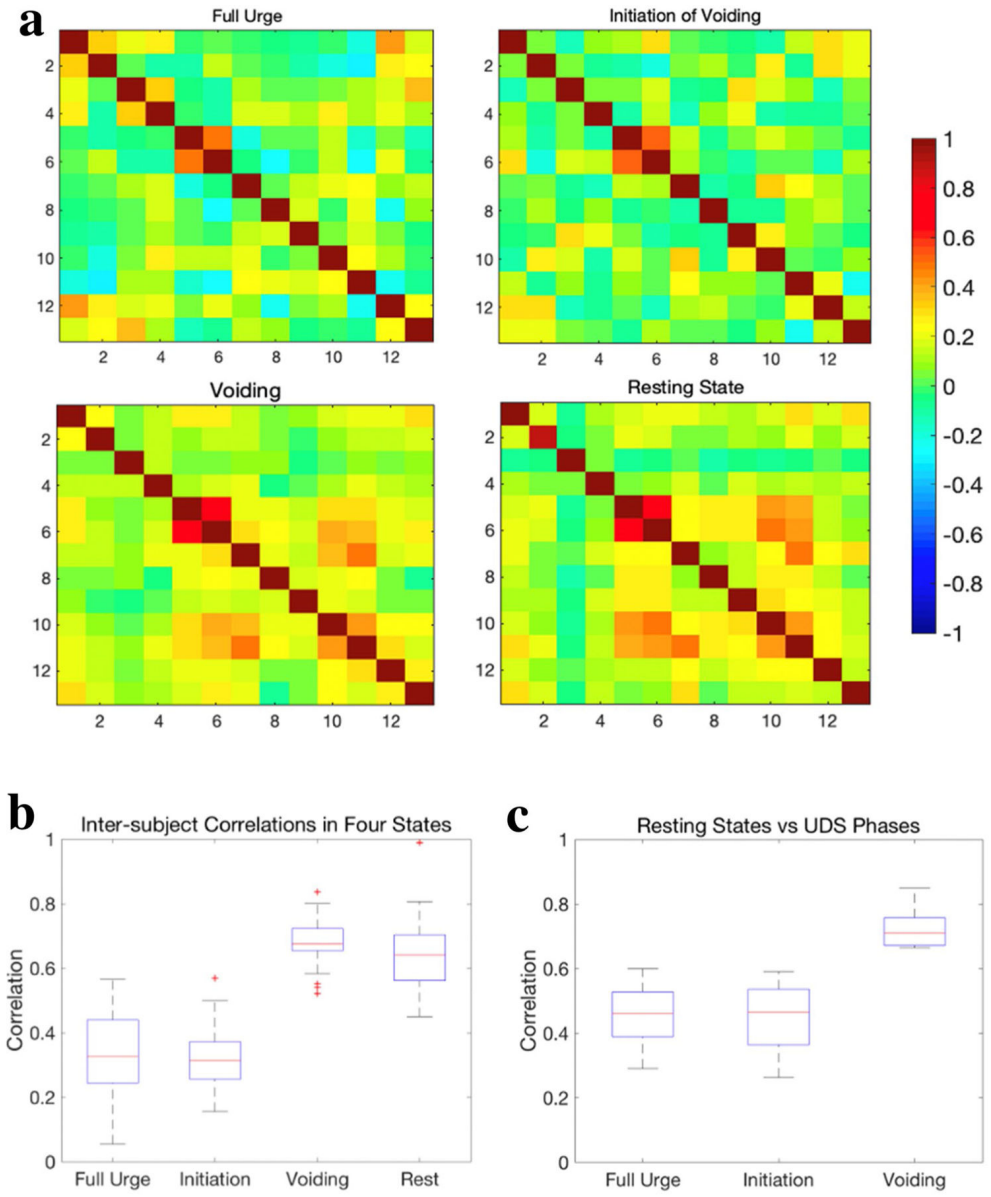
**a** Averaged functional connectivity matrices of all 13 ROIs for 9 subjects in the following three urodynamic phases ‘strong desire to void’, ‘initiation of voiding (or attempt at initiation of voiding)’, ‘voiding (or continued attempt of voiding)’, and resting states. The row and column represent each ROI number and their corresponding brain regions can be seen in Table 1. The color bar represents correlation values ranging from −1 to 1. **b** Group analysis of inter-subject correlations in functional connectivity within 13 ROIs across 9 subjects during three urodynamic phases and resting states, respectively. **c** The boxplot shows a group analysis of correlations within the bladder-related network between resting states and three urodynamic phases, respectively.

A good correlation (*r* > 0.5, *p* ≈ 0.1) between the medial and superior frontal gyrus in the right hemisphere was observed within all three urodynamic phases and resting states as shown in Fig. 1a. In addition, a moderate correlation (0.2 < *r* < 0.4, 0.31 < *p* < 0.14) was observed within IFG only at strong desire to void, not in any other states. During (attempt at) voiding initiation, a moderate correlation (*r* = 0.3, *p* = 0.19) between IFG and dlPFC in the right hemisphere was observed. Another moderate correlation was observed between SFG in the right hemisphere and SMA at the (attempt at) voiding initiation and remained strong (*r* = 0.3, *p* = 0.08) during (continued attempt of) voiding. Furthermore, this correlation was also found to be good (*r* = 0.5, *p* = 1e-8) in resting states. Conversely, a moderate correlation between the superomedial M1 and SMA at the initiation state decreased during (continued attempt of) voiding. During (continued attempt of) voiding, several brain regions within the bladder-related network were observed to work together to achieve the voiding function. This was not found to be the case at strong desire to void or (attempt at) voiding initiation. For example, the moderate correlation between SFG and the superomedial M1, and the moderate correlation between SFG and dlPFC were only observed during (continued attempt of) voiding. These additional correlations were also observed at resting states.

### Inter-subject similarities of the bladder-related network connections

To evaluate the subjects’ consistency in functional connectivity during three bladder-related urodynamic phases, we compared the inter-subject correlations across all subjects. As shown in Fig. 1b, the highest inter-subject correlations were observed during (continued attempt of) voiding. This result suggests that voiding is consistently achieved through similar bladder-related network connections. In addition, Fig. 1b also illustrates high correlations within 13 ROIs across all subjects in resting states. This indicates a good consistency of the intrinsic bladder-related network in the brain.

### Similarity in functional connectivity between urodynamic phases and resting states

We quantitatively compared the similarity in functional connectivity within 13 ROIs in all subjects during three urodynamic phases and during resting states. Figure 1c shows significantly higher correlations between resting states and the ‘(continued attempt of) voiding’ state than the correlations between resting state and ‘strong desire to void’ or ‘(attempt at) voiding initiation’. These results suggest that functional connectivity between the ROIs closely resembles resting states only during (continued attempt of) voiding.

## Discussion

This study aimed to investigate whether resting states can be utilized as a non-invasive alternative for specific phases of urodynamics in the brain’s bladder-related network. We quantitatively compared the functional connectivity within 13 ROIs belonging to the bladder-related network during three urodynamic phases and resting states using 7 T and 3 T fMRI data. We found that the resting-state intrinsic connectivity closely resembles the urodynamic functional connectivity of the bladder-related network only during (continued attempt of) voiding, and thereby concurrent urodynamic testing is still necessary for studying the effects at strong desire to void and voiding initiation.

### Implication for the functional connectivity during urodynamics and resting states

Similar to other studies, a good correlation between the medial and superior frontal gyrus in the right hemisphere was observed in all three urodynamics phases and resting states [11, 17, 18]. It has been reported that the supracallosal part of the medial frontal cortex is one of the micturition centers [7, 11, 19, 20]. These results suggest that the frontal lobe plays an important role in the regulation of micturition in both brain activations and functional connectivity. IFG was found to correlate with several ROIs, such as superomedial M1 and dlPFC, at strong desire to void and (attempt at) voiding initiation, but not during (continued attempt of) voiding or during resting states. This supports that IFG is involved in the attention network and decision-making since the activation of IFG has been reported both in subjects who could void successfully and those who were unable to void despite trying vigorously to do so [14, 21, 22].

Since MS is a heterogeneous disease that can affect multiple locations in the central nervous system, the difference in lesion burdens, locations, and sizes among each subject could affect individual bladder-related network differently to detect strong desire to void and subsequently initiate voiding (or attempt to do so) [23], resulting in the lower inter-subject correlation observed in these two states. The high correlations within the 13 ROIs across all subjects during (continued attempt of) voiding strongly suggest that voiding in female MS patients with VD is consistently achieved through similar network connections independent from the connections required for ‘strong desire to void’ and ‘(continued attempt at) voiding initiation’. An observed high inter-subject correlation in resting states indicates consistency within the individual bladder-related network.

### Resting state is an alternative for studying the voiding network

The highest correlations of functional connectivity within the bladder-related network were observed between resting states and the ‘(continued attempt of) voiding’ state in all subjects. Therefore, resting states can be utilized as a non-invasive alternative for investigating the voiding network. The major difference among all three urodynamic phases and resting states was that the superomedial M1 and SMA were more correlated within themselves and with other brain regions during (continued attempt of) voiding and resting states than at strong desire to void and initiation. Previous studies have reported that SMA is activated during contraction of the pelvic floor muscles and that they work together to provide protection against incontinence.[24-26] We hypothesize that when the pontine micturition center lifts the inhibition and the voiding reflex starts, the voiding continues with minimal activity changes, and thus the brain connectivity becomes similar to its resting states. Because the resting-state intrinsic connectivity resembles less the functional connectivity at strong desire to void and initiation compared to voiding, invasive concurrent urodynamic testing is still necessary for investigating brain networks at strong desire to void and voiding initiation.

### Limitations and future directions

Previous studies on supraspinal control of the bladder have consisted of various models and concurrent fMRI/UDS to examine bladder cycles, such as urine withholding [7-9], and pelvic floor contraction [10]. While these models can provide insight regarding brain activation throughout the bladder cycle, they are invasive and inherently do not reflect the natural voiding environment for participants, especially MS patients who may already strain to void. Our study compared the brain connectivity during task-based urodynamics and resting states using BOLD fMRI. We started by analyzing thirteen ROIs associated with the bladder storage and voiding phases (Table 1), which were reliably reported in previous publications by various groups. However, there are other brain regions that may be involved in the bladder cycle but not analyzed in the current study, such as the dorsal anterior/posterior cingulate cortex and putamen. Our future research direction is to investigate functional connectivity during urodynamics and resting states at a whole brain scale using an atlas. In addition, we will also explore the dynamic changes in resting-state functional connectivity using time-variant methods.[27-29] Lastly, the current subject population consisted of only female MS patients with VD. Although this patient group does not similarly reflect the brain activity in healthy individuals, and it is unclear whether our findings can be applied to other sex and patient populations. However, we believe this would provide a foundation for and encourage more future studies to look into brain–bladder network connectivity in neuropathic populations. We intend to study the similarity and difference in the brain’s bladder-related network between various patient populations, such as benign prostatic hyperplasia [30].

## Conclusion

Brain regions within the bladder-related network are highly correlated only during (continued attempt of) voiding. Additionally, the functional connectivity derived from a task-based urodynamics testing closely resembles the resting-state intrinsic connectivity only during (continued attempt of) voiding. These results were found to be consistent across all nine subjects and suggest that resting-state fMRI can be potentially utilized to reflect bladder-related brain networks during the voiding process but not the other states. Concurrent urodynamic/fMRI testing is still necessary for studying the effects of strong desire to void and (attempt at) voiding initiation.

## Supplementary Material

Supplementary material

## Figures and Tables

**Table 1:** Thirteen regions of interest associated with the bladder-related network

ROI #	MNI	Brodmann Area	Hemisphere	Area
1	52,24,12	44/45	Right	Inferior Frontal Gyrus (IFG)
2	63,18,18	44/45	Right	Inferior Frontal Gyrus (IFG)
3	40,35,6	46	Right	Inferior Frontal Gyrus (IFG)
4	48,4,10	6	Right	Inferior Frontal Gyrus (IFG)
5	6,−18,57	6	Right	Medial Frontal Gyrus (MFG)
6	3,−18,57	6	Right	Superior Frontal Gyrus (SFG)
7	2,2,62	6	Right	Superior Frontal Gyrus (SFG)
8	−12,−26,66	4	Left	Superomedial M1
9	12,−24,66	4	Right	Superomedial M1
10	−2,−16,68	6	Left	Supplementary Motor Area (SMA)
11	4,−6,64	6	Right	Supplementary Motor Area (SMA)
12	40,38,38	9	Right	Dorsolateral Prefrontal Cortex (dlPFC)
13	52,38,12	46	Right	Dorsolateral Prefrontal Cortex (dlPFC)

**Table 2:** Patient demographics and clinical variables

Patient Characteristics (N = 9)	Mean (range)
Age (years)	54.8 (35–77)
BMI (kg/m^2^)	26.9 (19.9–41.8)
MS duration (years)	14.7 (1–44)
%PVR/BC	45.9 (32.1–81.3)
Liverpool nomogram percentile	43.3 (5–96)
Performing CIC (%)	1/9 (11.1%)
DSD (%)	1/9 (11.1%)
Voided during fMRI (%)	3/9 (33.3%)
NDO during fMRI (%)	4/9 (44.4%)

BMI: body mass index; %PVR/BC: % post-void residual/bladder capacity; CIC: clean intermittent catheterization; NDO: neurogenic detrusor overactivity.
