# Supplementary material for: High spatial correlation in brain connectivity between micturition and resting states within bladder-related networks using 7 T MRI in multiple sclerosis women with voiding dysfunction
Source: World J Urol. 2021 Jan 29;39(9):3525–31. doi: 10.1007/s00345-021-03599-4 (PMC8344374; doi:10.1007/s00345-021-03599-4)
Supplement: Supplementary file 1 — Supplementary file1 (DOCX 381 KB) [file 345_2021_3599_MOESM1_ESM.docx]

**Appendix**

Example of detrusor overactivity (DO) on UDS report during concurrent UDS/fMRI examination in subject MS-304, marked with red box. Subject exhibited DO during urine withholding (after signaling that they felt full but before permission to void was given) during cycle 2 and 3 of the examination.

**
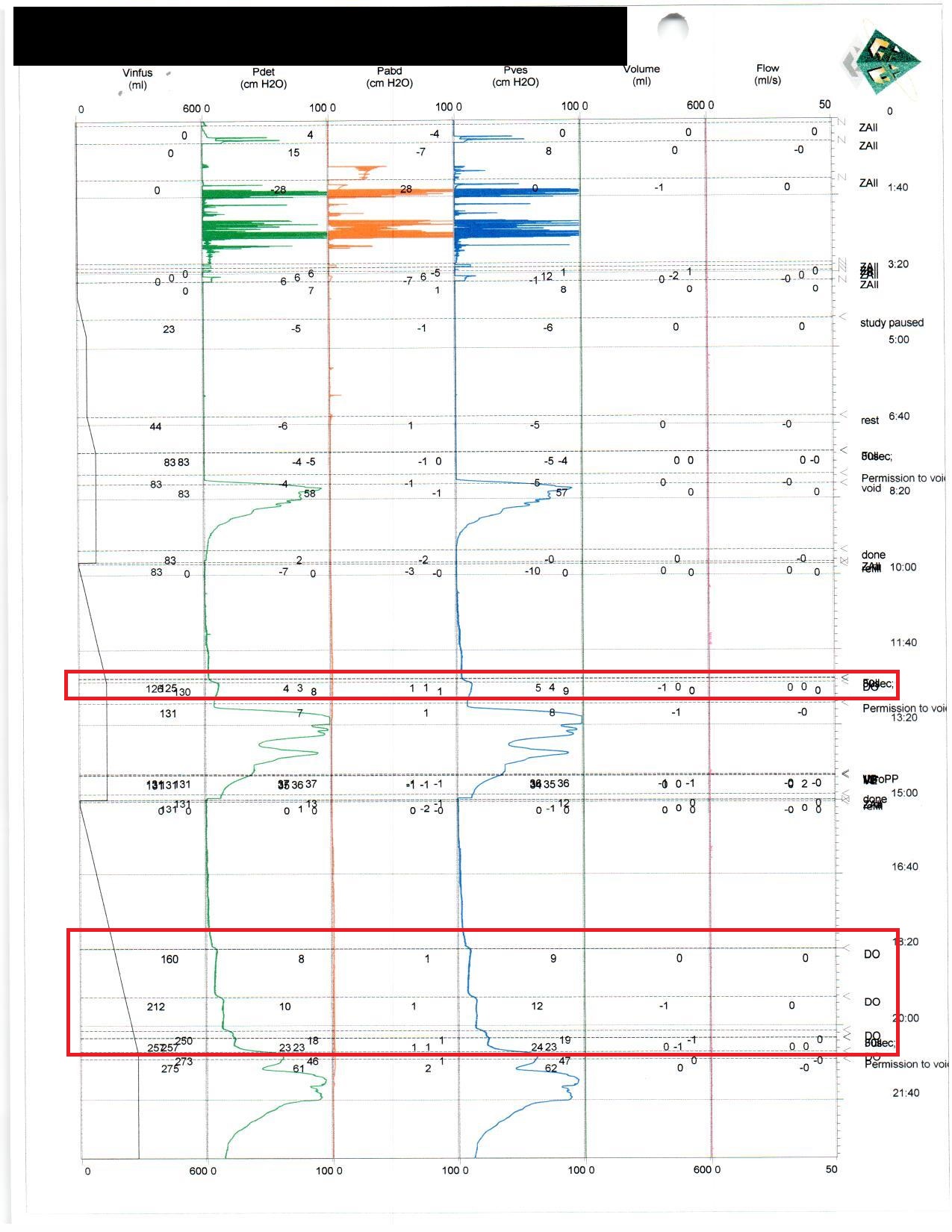
**
