# Supplementary material for: High spatial correlation in brain connectivity between micturition and resting states within bladder-related networks using 7 T MRI in multiple sclerosis women with voiding dysfunction
Source: World J Urol. Author manuscript; Available in PMC 2021 Oct 13. (PMC8344374; doi:10.1007/s00345-021-03599-4)

Appendix

Example of detrusor overactivity (DO) on UDS report during concurrent UDS/fMRI examination in subject MS-304, marked with red box. Subject exhibited DO during urine withholding (after signaling that they felt full but before permission to void was given) during cycle 2 and 3 of the examination.

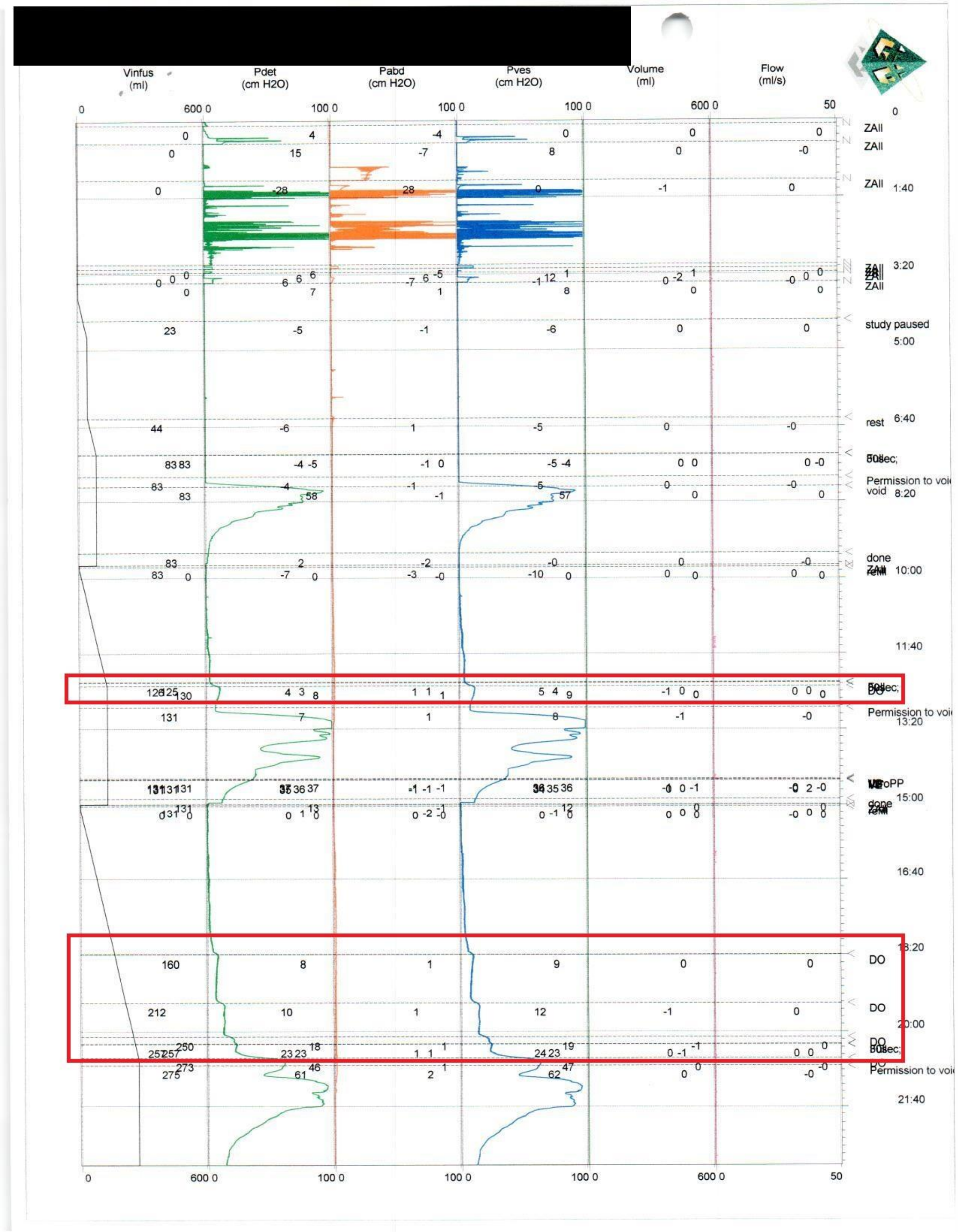

Supplement: Supplementary file 1 [file NIHMS1726073-supplement-Supplementary_material.pdf]
